# Genetically engineered biosynthetic pathways for nonnatural C_60_ carotenoids using C_5_-elongases and C_50_-cyclases in *Escherichia coli*

**DOI:** 10.1038/s41598-019-39289-w

**Published:** 2019-02-27

**Authors:** Ling Li, Maiko Furubayashi, Shifei Wang, Takashi Maoka, Shigeko Kawai-Noma, Kyoichi Saito, Daisuke Umeno

**Affiliations:** 1 Department of Applied Chemistry and Biotechnology, Chiba University, Chiba, Japan; 2 Research Institute for Production Development, Kyoto, Japan

## Abstract

While the majority of the natural carotenoid pigments are based on 40-carbon (C_40_) skeleton, some carotenoids from bacteria have larger C_50_ skeleton, biosynthesized by attaching two isoprene units (C_5_) to both sides of the C_40_ carotenoid pigment lycopene. Subsequent cyclization reactions result in the production of C_50_ carotenoids with diverse and unique skeletal structures. To produce even larger nonnatural novel carotenoids with C_50_ + C_5_ + C_5_ = C_60_ skeletons, we systematically coexpressed natural C_50_ carotenoid biosynthetic enzymes (lycopene C_5_-elongases and C_50_-cyclases) from various bacterial sources together with the laboratory-engineered nonnatural C_50_-lycopene pathway in *Escherichia coli*. Among the tested enzymes, the elongases and cyclases from *Micrococcus luteus* exhibited significant activity toward C_50_-lycopene, and yielded the novel carotenoids C_60_-flavuxanthin and C_60_-sarcinaxanthin. Moreover, coexpression of *M*. *luteus* elongase with *Corynebacterium* cyclase resulted in the production of C_60_-sarcinaxanthin, C_60_-sarprenoxanthin, and C_60_-decaprenoxanthin.

## Introduction

Carotenoids are a class of natural pigments covering yellow, orange and red colors. More than 750 carotenoids have been identified in various plants, fungi, and microorganisms^1^, and a wide range of essential biological functions have been described, with light-harvesting, photoprotection, antioxidatant, and pro-vitamin activities, and roles in the control of membrane fluidity^2^. In recent years, carotenoids have been increasingly considered in applications as therapeutic or bioelectronic materials^3,4^. Small changes in carotenoid structures have been associated with large differences in material properties^5^ and biological activities^3,6^. Hence, the discovery of the structurally novel carotenoids is eagerly awaited.

Most carotenoids have C_40_ backbones, although a few microbial carotenoids have C_30_ backbones. The structural diversity of carotenoids is mainly derived from differences in desaturation levels of the C_40_ backbone, types of end groups, and functional patterns of cyclization, oxidation, and esterification. Further structural diversity of carotenoids can be achieved by liberating carotenoids from their C_40_ backbones. For example, carotenoid cleavage enzymes can be used to produce a range of carotenoids with different-backbone sizes (C_14_, C_20_, or C_28_ carbons)^7^. These include precursors of bixin (C_24+1_), crocetin (C_20_), retinals (C_20_), and other hormonal compounds such as strigolactones (C_13_) and abscisic acid (C_15_), as reviewed previously^3^.

Another source of structure and size diversity comes from the attachment of additional isoprene units (C_5_) to C_40_ carotenoids. Since C_45_ and C_50_ carotenoids were first reported in the 1960s^8,9^, many of these carotenoids have been identified in prokaryotes, including gram-positive bacteria such as *Micrococcus*^10^, *Corynebacterium*^11^, *Dietzia*^12^, and extreme halophilic archaea such as *Halobacterium* or *Haloarcula*^13^. Although the natural functions of these carotenoids are not fully understood, contributions to cell membrane fluidity and permeability have been suggested^13^. In addition, the attachment of C_5_ units reportedly confers remarkable antioxidant activities to carotenoids^14^, and the resulting compounds have been considered for their potential as therapeutic agents^13–15^.

**Figure 1:**
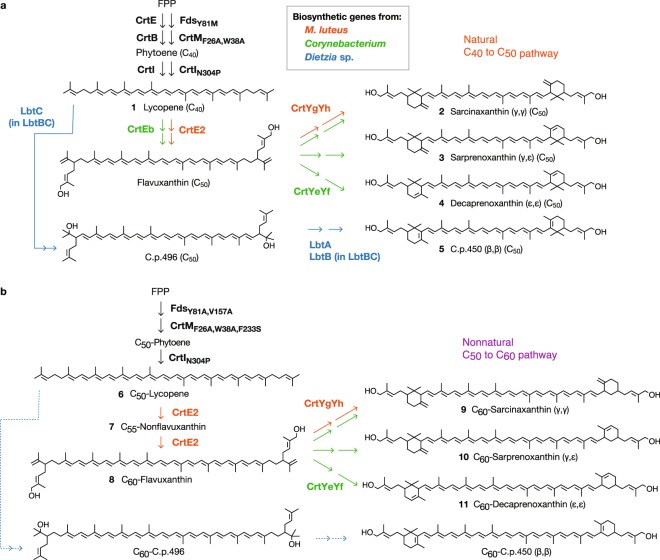
C_5_-elongation and cyclization pathways for natural (C_50_) and nonnatural (C_60_) carotenoids. (**a**) Natural C_40_-to-C_50_ carotenoid pathway. Using lycopene (C_40_) as a substrate, the lycopene elongases CrtEb from *Corynebacterium* and CrtE2 from *M*. *luteus* attaches two isoprene units to C_40_. The resulting flavuxanthin (C_50_) is then cyclized by the γ- and/or ε-cyclases CrtYe/Yf (*Corynebacterium*) or CrtYg/Yh (*M*. *luteus*) to produce sarcinaxanthin, sarprenoxanthin, or decaprenoxanthin (all C_50_). LbtABC genes from *Dietzia* sp. CQ4 produces the β,β-cyclic C_50_ carotenoid C.p.450 via the independent intermediate C.p.496. (**b**) Nonnatural C_50_-to-C_60_ carotenoid pathway construction. Combined expression of *Corynebacterium* and *M. luteus* elongases and cyclases resulted in the conversion of laboratory-generated C_50_-lycopene to C_60_ carotenoids with γ and/or ε-cyclic ends. We could not obtain C_60_ counterparts of β-end C_50_ carotenoids (indicated in arrows with dashed lines).

To date, four natural biosynthetic pathways for C_50_ carotenoids has been reported^10–12,16^. All known C_50_ carotenoids are derived from lycopene, a four-step enzymatic desaturation product of phytoene, which is produced by the condensation of two molecules of geranylgeranyl diphosphate (C_20_). One pathway from extremely halophillic archaea *Haloarcula japonica* yields acyclic C_50_ carotenoid bacterioruberin^16^ as the final product, whereas three others produce cyclic C_50_ carotenoids (Fig. 1a). In the *Micrococcus luteus* carotenoid biosynthetic pathway, lycopene elongase (CrtE2) attaches two additional isoprene units (DMAPP) to the 2,2′-position of lycopene to produce the acyclic C_50_ carotenoid flavuxanthin, and subsequent γ-cyclization by cyclase (CrtYgYh) produces sarcinaxanthin (γ,γ-ring)^10^. C_50_ carotenoid synthesis in *Corynebacterium* also proceeds via the flavuxanthin intermediate, but the expressed cyclase (CrtYeYf) is less specific and yields a mixture of decaprenoxanthin^10,11^ (ε,ε-ring), sarprenoxanthin^10^ (ε,γ-ring) and sarcinaxanthin, when expressed in *E*. *coli*. In contrast, the bacterium *Dietzia*, reportedly produces C_50_ carotenoids with β,β-rings (C.p.450)^12^ via a different acyclic intermediate (Fig. 1a).

In a previous study, we constructed a nonnatural C_50_ backbone carotenoid pathway in which two geranylfarnesyl diphosphates (C_25_PP) are condensed to produce C_50_-phytoene (Fig. 1b). This compound is subsequently subjected to a six-step desaturation reaction resulting in the synthesis of C_50_-lycopene. Using a metabolic filtering approach^17^, we extended this C_50_-lycopene pathway to specifically produce the nonnatural C_50_ backbone carotenoids C_50_-β-carotene, C_50_-zeaxanthin, C_50_-canthaxanthin, and C_50_-astaxanthin. In this study, we systematically expressed elongase and cyclase in engineered *E*. *coli* strains harboring enzymes of the C_50_-lycopene synthetic pathway and established pathways for the synthesis of the novel carotenoids C_60_-flavuxanthin, C_60_-sarcinaxanthin, C_60_-sarprenoxanthin, and C_60_-decaprenoxanthin (Fig. 1b).

## Results

### Activities of elongase and cyclase in the natural C_40_ pathway

We selected *Corynebacterium glutamicum*, *M*. *luteus*, and *Dietzia* sp. CQ4 as sources of lycopene elongases and C_50_ cyclases, because the C_50_ carotenoid pathways from these organisms have been previously reconstructed in *E*. *coli*^10–12^. The carotenoid cluster (shown in Supplementary Fig. 1) of *C*. *glutamicum*^11^ comprises *crtEb* (lycopene elongase), *crtYeYf* (heterodimeric C_50_ ε/γ-cyclase), and lycopene-producing genes (*crtEBI*). The expression of this gene cluster in *E*. *coli* produces sarprenoxanthin, decaprenoxanthin, and sarcinaxanthin via the acyclic intermediate flavuxanthin^10,11^ (Fig. 1a). The corresponding gene cluster in *M*. *luteus*^10^ encodes *crtE2* (lycopene elongase) and *crtYgYh* (C_50_ γ-cyclase), and coexpression of these genes with *crtEBI* results in specific accumulation of sarcinaxanthin^10^. Uniquely, the carotenogenic gene cluster of *Dietzia sp*. CQ4^12^ encodes a gene for elongase (*lbtC*) that is fused in frame with a single subunit of cyclase (*lbtB*), which forms a heterodimer with another unit of cyclase (*lbtA*). Expression of *lbtB* with an *lbtA* gene product results in the production of a functional C_50_ β-cyclase^12^.

**Figure 2:**
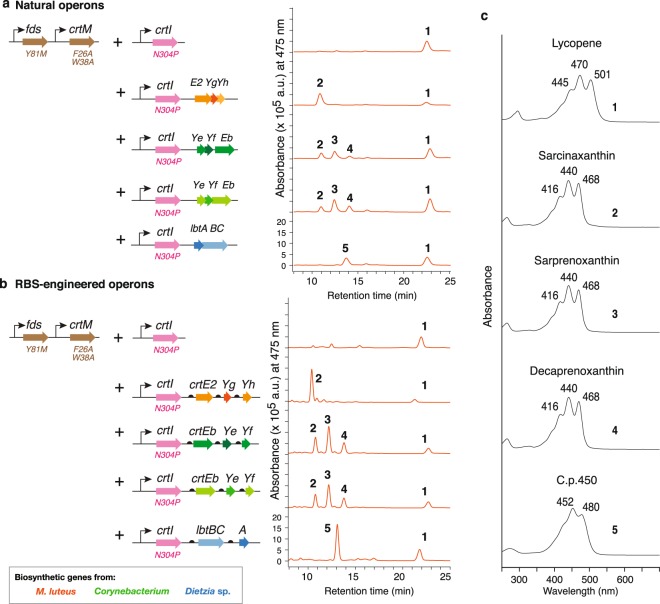
Lycopene elongases and C_50_ cyclases function in the natural C_40_-to-C_50_ pathway. HPLC chromatograms of carotenoid extracts from *E*. *coli* cells harboring plasmids with genome-derived natural gene cluster (**a**) or RBS-redesigned artificial operons (**b**), together with lycopene biosynthetic genes. Peak numbers correspond with those indicated in c and Fig. 1. (**c**) Absorbance spectra of indicated compounds.

Although different in organization, all of the elongase and cyclase genes are clustered in small <1.7-kb regions (Supplementary Fig. 1), allowing one-step PCR amplification from genomic DNA. We cloned these DNA fragments into a plasmid that encodes *Pantoea ananatis* phytoene desaturase variant^17^ *crtI*_*N304P*_, a variant that can desaturate both (C_40_-) phytoene and C_50_-phytoene (see Fig. 2a for plasmid construct). In addition to these three operons (for *C*. *glutamicum*, *M*. *luteus* and *Dietzia* sp.), we cloned the elongase and cyclase genes from *Corynebacterium efficiens*. The carotenoid gene cluster of this organism has exactly the same organization as that of *C*. *glutamicum*, and in our homology analyses, DNA sequence identity of the two clusters (from *crtE* to *crtEb*, see Supplementary Fig. 1) was 64.3%. Yet, the functions of the *C*. *efficiens* carotenoid pathway and its products remain uncharacterized.

To characterize the functions of elongase and cyclase in the context of natural (C_40_) pathway (Fig. 1a) in *E*. *coli*, we used an engineered *E*. *coli* strain that constitutively expresses *fds*_*Y81M*_ and *crtM*_*F26A*,*W38A*_ genes (*crtEB* equivalent^17^) and produces phytoene (C_40_). We introduced plasmids encoding phytoene desaturase (a *crtI* variant), elongases and cyclases into this strain, and after culturing for 48 h, we extracted carotenoids using acetone and analyzed these using high performance liquid chromatography (HPLC) (Fig. 2a). These analyses showed that cells expressing *C*. *glutamicum crtYeYfEb* produced a mixture of (C_50_-) sarcinaxanthin (**2**), (C_50_-) sarprenoxanthin (**3**), and (C_50_-) decaprenoxanthin (**4**), with significant quantities of their substrate (C_40_-) lycopene (**1**). Cells expressing *C*. *efficiens crtYeYfEb* indistinguishable composition of carotenoids as those from the *C*. *glutamicum* gene cluster. Cells expressing *M*. *luteus crtE2YgYh* produced a single peak of (C_50_-) sarcinaxanthin (**2**), whereas expression of *Dietzia* sp. CQ4 *lbtABC* resulted in the specific production of C.p.450 (C_50_). Despite the differences in the source of lycopene biosynthetic genes, promoters, RBS sequences, *E*. *coli* strain or culture condition, product distribution of the pathway we constructed was very similar to those from previous reports^10^.

To determine whether these enzymes can metabolize C_50_-lycopene, we coexpressed plasmids encoding the elongase and cyclase enzymes in another *E*. *coli* strain that constitutively expresses *fds*_*Y81A*,*V157A*_ and *crtM*_*F26A*,*W38A*,*F233S*_ and produces C_50_-phytoene^17^. In this strain, the phytoene desaturase mutant (CrtI_N304P_) can desaturate C_50_-phytoene to produce C_50_-lycopene selectively^17^, providing a sole substrate for the elongase and cyclases in this stain. However, we observed no novel peaks but C_50_-lycopene in carotenoid fractions from this strain.

### Ribosome binding site (RBS)-optimized cyclase/elongase results in higher production of cyclic C_50_ carotenoids

In the previous section, we observed (C_40_-) lycopene accumulated in cells expressing elongase and cyclases (Fig. 2a), suggesting the presence of inefficiencies of this pathway, and room for improvement. The present four carotenoid operon constructs share similar operon organization, in which open reading frames (ORF) for cyclases and elongases are partially overlapping. Specifically, the start codon of *M*. *luteus crtYg* is 28 base pairs upstream of the stop codon of the *crtE2* gene, and the start codon of *crtYh* overlaps with the stop codon of the *crtYg* gene. In *C*. *glutamicum* and *C*. *efficiens*, *crtYe* and *crtYf* overlap by 4 bases, and in *Dietzia*, *lbtA* and *lbtBC* genes also overlap by 4 bases, as is commonly observed in various gene clusters containing carotenoid operons^10^. We investigated translation initiation rates using the RBS calculator^18–20^ and found RBS scores as low as 0.01 (Table 1), suggesting very low translation initiation rates and likely formation of stable secondary mRNA structures. In the native operons with overlaps in translational stop and start of ORFs, the translational re-initiation is likely ensuring efficient translation *in vivo*.

**Table 1:** Ribosome binding site strengths of original and designed operons.

Organism	Gene	RBS strength in v1 plasmid	Designed RBS strength in v2 plasmid
*M*. *luteus*	*crtE2*	812	4296
	*crtYg*	0.01	1525
	*crtYh*	140	4700
*Dietzia sp*. *CQ4*	*lbtA*	968	4106
	*lbtBC*	15	4700
*C*. *glutamicum*	*crtYe*	4493	3891
	*crtYf*	4342	5886
	*crtEb*	389	5066
*C*. *efficiens*	*crtYe*	776	1113
	*crtYf*	33	2663
	*crtEb*	93	1990

RBS strength obtained using a ribosome binding site (RBS) calculator^18–20^. The RBS and open reading frame (ORF) sequences are listed in Supplementary Tables S1 and S2.

To improve expression levels of elongases and cyclases in *E*. *coli*, we redesigned our artificial operons (Fig. 2b) by (1) separating the ORFs to yield tandem and distinct non-overlapping reading frames, and (2) to provide stronger RBSs, designed using RBS calculator to have RBS strengths between 1,000 - 5,000 (Table 1). These RBS strengths range were chosen since it was previously confirmed optimal for expressing various carotenoid biosynthetic genes in the plasmid backbone we use in this study^17,21^ (i.e. p15A, pUC). When expressed with (C_40_-) lycopene pathways, all of these new constructs with engineered RBSs led to improved production of natural C_50_ carotenoids and significant reductions in unconverted lycopene contents (Fig. 2b).

### Production of nonnatural cyclic C_60_ carotenoids

**Figure 3:**
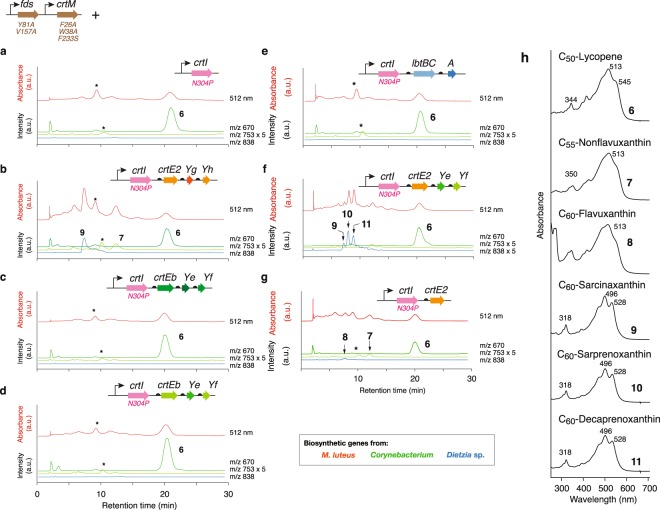
Lycopene elongases and C_50_ cyclases function in the C_50_-to-C_60_ pathway. (**a**–**g**) HPLC chromatogram of carotenoid extracts from *E*. *coli* cells expressing genes for C_50_-phytoene production with indicated genes. The indicated peak numbers correspond with those in Fig. 1. Peaks labelled with asterisks correspond to unidentified non-carotenoid compounds. (**h**) Absorbance spectra of the indicated peaks.

Following the RBS engineering of elongases and cyclases, we cotransformed the new constructs into the strains that selectively synthesize C_50_-phytoene (Fig. 3). From the cells expressing *M*. *luteus crtE2YgYh*, we observed novel peaks **7** and **9** in HPLC chromatograms, and these were eluted earlier than that of C_50_-lycopene (**6**) (Fig. 3a,b). Peak **9** exhibited absorbance maxima at 496 nm; shorter than that for C_50_-lycopene (**9**, 512 nm). The m/z value (837) of peak **9** matched that of C_60_-sarcinaxanthin (C_60_H_84_O_2_, Fig. 1), and the carotenoid from peak **9** had absorption maxima at 460, 494, and 527 nm (Fig. 3h). Furthermore, analyses using positive ion electrospray ionization time-of-flight mass spectrometry (ESI TOF MS) at *m/z* 837.6542 MH^+^ indicated a molecular formula for this carotenoid of C_60_H_84_O_2_ (C_60_H_85_O_2_ calcd. for 837.6550) (Supplementary Fig. 2). The structure of this carotenoid was determined as C_60_-sarciniaxanthin using nuclear magnetic resonance (^1^H-NMR) with correlation spectroscopy (COSY) and rotating frame overhause effect spectroscopy (ROESY) (Table 2). In addition, we identified peak **7** as the C_60_ pathway intermediate C_55_-nonflavuxanthin based on an absorbance maximum of 513 nm and a m/z value of 753 (C_55_H_76_O).

**Table 2:** ^1^H NMR data for C_60_-sarcinaxanthin and C_60_-flavuxanthin in CDCl_3_.

Position	C_60_-Sarcinaxanthin (9)			Position	C_60_-Flavuxanthin (8)		
	d	Mult.	J (Hz)		d	Mult.	J (Hz)
H-2 (2′)	1.28	m		H-2 (2′)	2.08	m	
H-3 (3′)	1.18	m		H_2_-3 (3′)	1.56	m	
	1.71	m					
H-4 (4′)	2.05	m		H_2_-4 (4′)	2.00	m	
	2.35	m					
H-6 (6′)	2.48	d	10	H-6 (6′)	5.93	d	11
H-7 (7′)	5.83	d	15.5, 10	H-7 (7′)	6.48	dd	15, 11
H-8 (8′)	6.12	d	15.5	H-8 (8′)	6.24	d	15
H-10 (10′)	6.12	d	12	H-10 (10′)	6.18	d	12
H-11 (11′)	6.62	dd	15, 12	H-11 (11′)	6.63	dd	15, 12
H-12 (12′)	6.34	d	15	H-12 (12′)	6.36	d	15
H-14 (14′)	6.23	d	11	H-14 (14′)	6.23	d	11
H-15 (15′)	6.64	dd	15, 11	H-15 (15′)	6.64	dd	15, 11
H-16 (16′)	6.38	d	15	H-16 (16′)	6.38	d	15
H-18 (18′)	6.27	br. d	10	H-18 (18′)	6.27	br. d	10
H-19 (19′)	6.64	m		H-19 (19′)	6.64	m	
H_3_-20 (20′)	0.96	s		H-20 (20′)	4.70	br. S	
				H-20 (20′)	4.78	br. S	
H_3_-21 (21′)	0.73	s		H_3_-21 (21′)	1.63	s	
H-22 (22′)	4.53	s		H_3_-22 (22′)	1.80	s	
H-22 (22′)	4.76	s					
H_3_-23 (23′)	1.98/1.99	s		H_3_-23 (23′)	1.97	s	
H_3_-24 (24′)	1.98/1.99	s		H_3_-24 (24′)	1.98/1.99	s	
H_3_-25 (25′)	1.98/1.97	s		H_3_-25 (25′)	1.98/1.97	s	
H-26 (26′)	1.72	m					
H-26 (26′)	2.24	dd	14, 5.5	H_2_-26 (26′)	2.10	m	
H-27 (27′)	5.43	m		H-27 (27′)	5.36	t	5
H_3_-29 (29′)	1.67	s		H_3_-29 (29′)	1.67	s	
H_2_-30 (30′)	4.03	s		H_2_-30 (30′)	4.00	s	

See Supplementary Fig. S4 for the numbering of carotenoid structure.

The *crtE2* gene expression (without *crtYgYh*) from *M*. *luteus* resulted in a new peak **8** with an m/z value of 838 and an absorbance maximum of 513 nm (Fig. 3g). The carotenoid from peak **8** showed absorption maxima at 460, 494, and 527 nm (Fig. 3h). Positive ion ESI TOF MS at *m/z* 857.6107 M + Na^+^ revealed the molecular formula C_60_H_82_O_2_ (C_60_H_82_O_2_Na calcd. for 857.6212) (Supplementary Fig. 3) for this carotenoid, and structural analyses using ^1^H-NMR with COSY and ROESY revealed the carotenoid C_60_-flavuxanthin (Table 2).

We did not observe any novel carotenoid peaks in experiments using elongase and cyclase genes from *C*. *glutamicum*, *C*. *efficiens*, or *Dietzia* sp. CQ4 (Fig. 3c–e). Hence, the lycopene elongases (CrtEb in *Corynebacterium* and LbtC in *Dietzia* sp. CQ4) at least fail to convert C_50_-lycopene in *E*. *coli*. However, the activities of the cyclases (CrtYeYf from *Corynebacterium* and LbtAB from *Dietzia* sp. CQ4) were still unknown, since the first elongase step failed to provide the substrate (C_60_-flavuxanthin or C_60_-C.p.496) for these cyclase enzymes.

Because *Corynebacterium* and *M*. *luteus* pathways share the intermediate flavuxanthin, we generated a chimera operon containing *M*. *luteus crtE2* and *C*. *efficiens crtYeYf*. Following expression in cells, we detected three carotenoid peaks (Fig. 3f) (**9**, **10**, and **11**) with identical absorption spectra (Fig. 3h), indicating the presence of the same chromophore. Although we could not determine the NMR spectroscopy of the compound from the associated chromatographic peaks, their absorption spectra, m/z values (838) and retention times strongly indicate that peaks **9**, **10**, and **11** were C_60_-sarcinaxanthin (as shown in Fig. 3b), C_60_-sarprenoxanthin, and C_60_-decaprenoxanthin, respectively. These results also indicate that cyclases (*crtYeYf)* from *Corynebacterium* are functional in the C_50_-to-C_60_ pathway.

## Discussion

In this study, we demonstrated that carotenoid elongation and cyclization enzymes from natural C_50_ carotenoid pathways metabolize C_50_-lycopene to novel nonnatural C_60_ carotenoids. These carotenoids (C_60_-flavuxanthin, C_60_-sarcinaxanthin, C_60_-sarprenoxanthin, and C_60_-decaprenoxanthin) are larger than any known natural carotenoids, and their absorbance spectra are red-shifted by as much as 58 nm compared with the natural counterpart (440 nm vs. 496 nm). With rare γ-ring structures, these C_60_ carotenoids provide a unique set of accessible carotenoid structures with as yet unknown functions. Natural C_50_ carotenoids were previously discovered in thicker membranes of halophillic bacteria, which survive in extreme hypersaline and low-temperature environments^13,14,16^. The present nonnatural C_60_ carotenoids are interesting candidates for functional characterization in extremophiles. These future studies may lend understanding to the roles of long-chain carotenoids in nature.

Through the coexpression of natural carotenoid enzymes, we have increased the number of laboratory-generated C_50_ carotenoid pathways. Many carotenoid enzymes from C_40_ and C_30_ pathways exhibit significant substrate promiscuity and accept a wide range of substrates with recognizable locally specific^22^ structures. Consequently, these enzymes are active in nonnatural pathway contexts^22–25^ without any mutations. However, there is increasing reports on carotenoid modifying enzymes that exhibit unexpected selectivity against non-cognate but very similar substrates. For example, lycopene ε-cyclases from plants (LCYe) cyclize only one end of the acyclic substrate lycopene and leave the other end un-cyclized^26^. Hence, ε-cyclases likely possess mechanisms for avoiding cyclization of non-cognate substrates. Similarly, the β-carotene 15,15′ cleavage enzyme BCMO1 only accepts β-carotene (β,β-end) as a substrate and does not act on ε-carotene (ε,ε-end). However, after removing the ε-end via 9′,10′ cleavage by BCMO2, BCMO1 precisely cleaves the 15,15′ bond and liberates retinal^27^.

While the elongases from *Corynebacterium* and *Dietzia* sp. CQ4 exhibited a significant activity towards natural substrate (C_40_-lycopene), they did not show any activity towards C_50_-lycopene. Considering that enzymes from both strains have significant activity in the C_40_-to-C_50_ context, it is unlikely that their inability to act on C_50_-lycopene reflects a lack of activity in *E*. *coli*. The more likely alternative is that some of these enzymes have higher substrate/size specificity and resist C_50_-lycopene as a substrate. Kim *et al*. showed that the *Corynebacterium* elongase CrtEb is functional in another non-cognate context and acts on the ψ-end of the C_30_ carotenoid 4,4′-diaponeurosporene^25^. Given this unpredictability of promiscuous functions toward non-cognate substrates, studies of several accessible gene candidates are required to identify genes that perform intended nonnatural tasks. Also, it is interesting how or whether *Dietzia* elongase and cyclase and *Corynebacterium* elongase, which were found non-functional in C_50_-to-C_60_ context in the present work, can acquire these new activities by mutations.

## Methods

### Strains and reagents

*E*. *coli* XL10-Gold cells were used for cloning, and XL1-Blue cells were used for carotenoid production. All enzymes were purchased from New England Biolabs. Lennox-LB Broth Base was purchased from Life Technologies, Bacto^TM^ Yeast Extract, and Bacto^TM^ Tryptone were purchased from BD Biosciences, and all other chemicals and reagents were obtained from Nacalai Tesque (Kyoto, Japan). The antibiotics carbenicillin and chloramphenicol were used at 30 and 50 µg/mL, respectively.

### Plasmid construction

pUCara-crtI_N304P_, a plasmid encoding a *crtI*_*N304P*_ gene downstream of an arabionse inducible *araBAD* promoter, was derived from a previous study^17^. The plasmids pUCara-crtI_N304P_-crtYeYfEb_Cg_, pUCara-crtI_N304P_-crtYeYfEb_Ce_, pUCara-crtI_N304P_-crtE2YgYh_Ml_, and pUCara-crtI_N304P_-lbtABC_CQ4_ were constructed by amplifying genes for elongase and cyclase from various genomes using the primers listed in Supplementary Table 1 and cloning these into the ApaI/SpeI restriction site of pUCara-crtI_N304P_. The plasmids pAC-fds_Y81A,V157A_-crtM_F26A,W38A,F233S_ and pAC-fds_Y81M_-crtM_F26A,W38A_ were derived from the previous study^17^. The *fds* and *crtM* variant genes on these plasmids were expressed constitutively under *lac* promoter. Supplementary Table 2 shows the DNA sequence of the designed RBS construct. These sequences were concatenated and were then inserted into the ApaI/SpeI restriction site of pUCara-crtI_N304P_ without spacer sequences.

### Culture conditions

Single colonies were inoculated into 2 ml of LB media with antibiotics in culture tubes and were shaken at 37 °C for 16 h. Overnight cultures of 2 mL were diluted 100-fold into 40 mL of fresh Terrific Broth media in 200 mL flasks and were then shaken at 200 rpm in an incubator at 30 °C. After 8 h, 0.2% (w/v) arabinose inducer was added and cells were cultured for an additional 40 h.

### Product extraction and purification

Cell cultures were centrifuged at 3,270 × *g* for 15 min at 4 °C. Cell pellets were washed with 10 ml of 0.9% (w/v) NaCl_aq_ and were then repelleted by centrifugation. Products were extracted by vigorously vortexing for 5 min in 10 ml of acetone containing 30-mg/L butylated hydroxytoluene. One-mL aliquots of hexane and 35-ml aliquots of 1% (w/v) NaCl_aq_ were then added, and the samples were centrifuged at 3,250 × *g* for 15 min. After collecting the product-containing hexane phase, the solvent was evaporated in a vacuum concentrator. Extracts were finally dissolved in 15–50-μl aliquots of tetrahydrofuran:methanol (6:4) for further analysis.

### HPLC–MS analysis for compound identification and quantification

Aliquots (2–15 μL) of final extracts were analyzed using Shimadzu Prominence HPLC system equipped with LCMS-2020 MS spectrometer, a photodiode array (PDA) detector with Waters Spherisorb ODS2 Analytical Columns (4.6 × 250 mm, 5 µm, PSS831915). Mobile phases comprised acetonitrile/tetrahydrofuran/methanol (58:7:35) at a flow rate of 2 mL/min (Fig. 2a), acetonitrile/tetrahydrofuran/methanol (58:4:38) at a flow rate of 1 mL/min (Fig. 2b), and acetonitrile/tetrahydrofuran/methanol (30:7:63) at a flow rate of 1.5 mL/min (Fig. 3). Elutes were detected using PDA (200–700 nm) and APCI-MS. Mass scans were performed from *m*/*z* 10 to *m*/*z* 1000 with a 300 °C interface temperature, a 300 °C DL, a ± 4500-V interface voltage, and a neutral DL/Qarray with N_2_ as the nebulizing gas.

ESI TOF MS spectra were acquired using a Waters Xevo G2S Q TOF mass spectrometer (Waters Corporation, Milford, CT, USA) equipped with an Acquity UPLC system with scanning from *m/z* 100 to 1,500 with a capillary voltage of 3.2 kV, a cone voltage of 40 eV, and a source temperature of 120 °C. Nitrogen was used as the nebulizing gas at a flow rate of 30 L/h. MS/MS spectra were measured using a quadrupole-TOF MS/MS instrument with argon as a collision gas at a collision energy of 30 V. ^1^H NMR (500 MHz) including COSY and ROESY spectra were generated using a Varian UNITY INOVA 500 spectrometer in CDCl_3_ with tetramethylsilane as an internal standard.

## Supplementary information

Dataset 1

## Data Availability

The datasets generated during and/or analysed during the current study are available from the corresponding author on reasonable request.
