# Supplementary material for: Genetically engineered biosynthetic pathways for nonnatural C60 carotenoids using C5-elongases and C50-cyclases in Escherichia coli
Source: Sci Rep. 2019 Feb 27;9:2982. doi: 10.1038/s41598-019-39289-w (PMC6393565; doi:10.1038/s41598-019-39289-w)
Supplement: Supplementary file 1 — Dataset 1 [file 41598_2019_39289_MOESM1_ESM.docx]

**Supplementary Information for**

**Genetically engineered biosynthetic pathways for nonnatural
C_60_ carotenoids using C_5_-elongases and C_50_-cyclases in *Escherichia coli*.**

Ling Li^§1^, Maiko Furubayashi^§1^, Shifei Wang^1^, Takashi Maoka^2^,
Shigeko Kawai-Noma^1^, Kyoichi Saito^1^, Daisuke Umeno*^1^

^1^Department of Applied Chemistry and Biotechnology, Chiba University, Japan

^2^Research Institute for Production Development, Japan

^§^Both authors contributed equally to this work.

*Corresponding author:

Daisuke Umeno, ^1^Department of Applied Chemistry and Biotechnology, Chiba University, Japan

umeno@faculty.chiba-u.jp


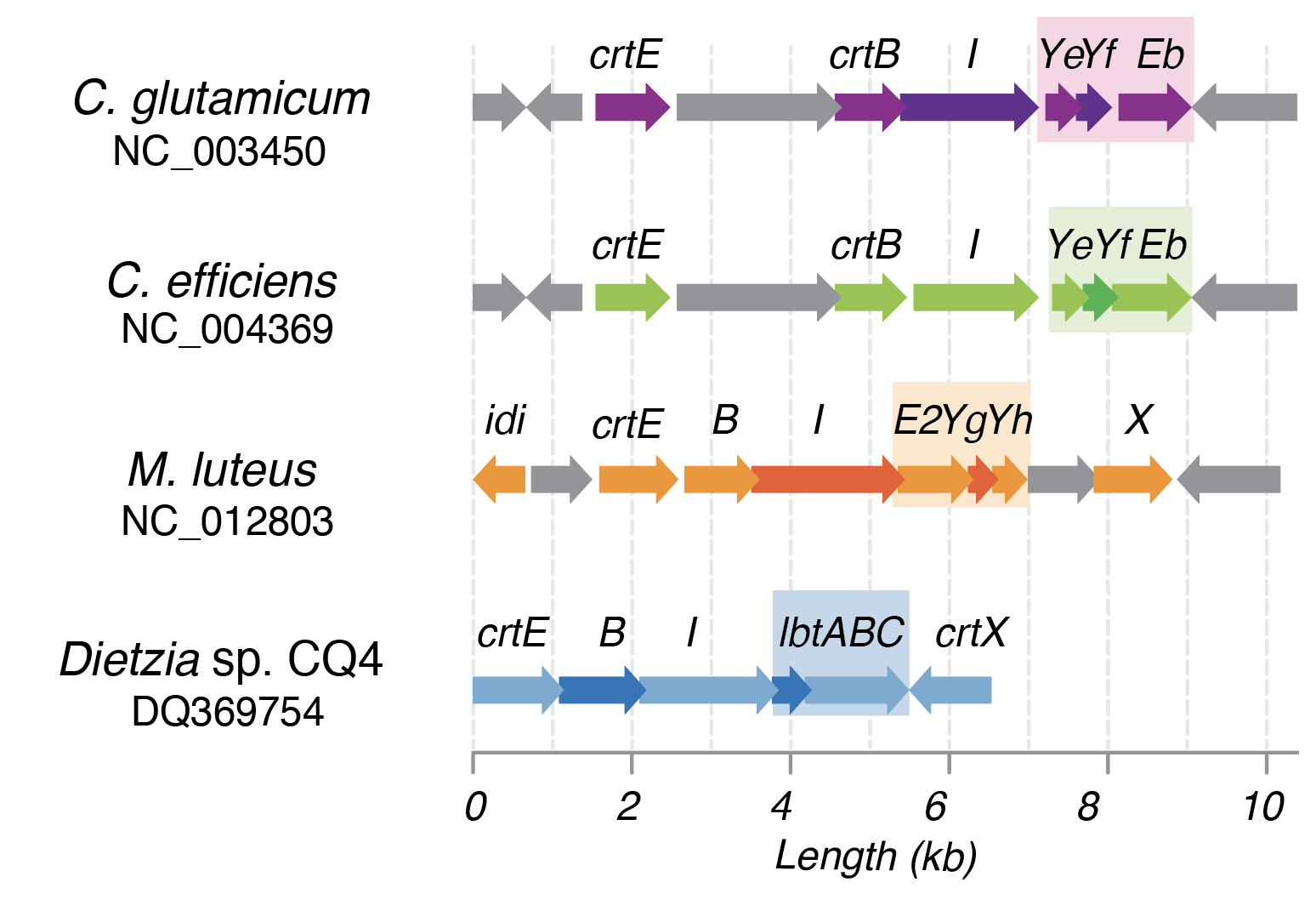


**Supplementary Figure 1.** Carotenoid gene cluster from different organisms.

Genbank accession numbers are shown below the names of the organisms. Carotenoid-pathway related enzymes are indicated in colors, while the unrelated or unknown genes are indicated in grey.

**Supplementary Figure 2.** Mass spectrum and absorbance spectrum for C_60_-sarcinaxanthin (ESI+, ESI-, MS/MS)


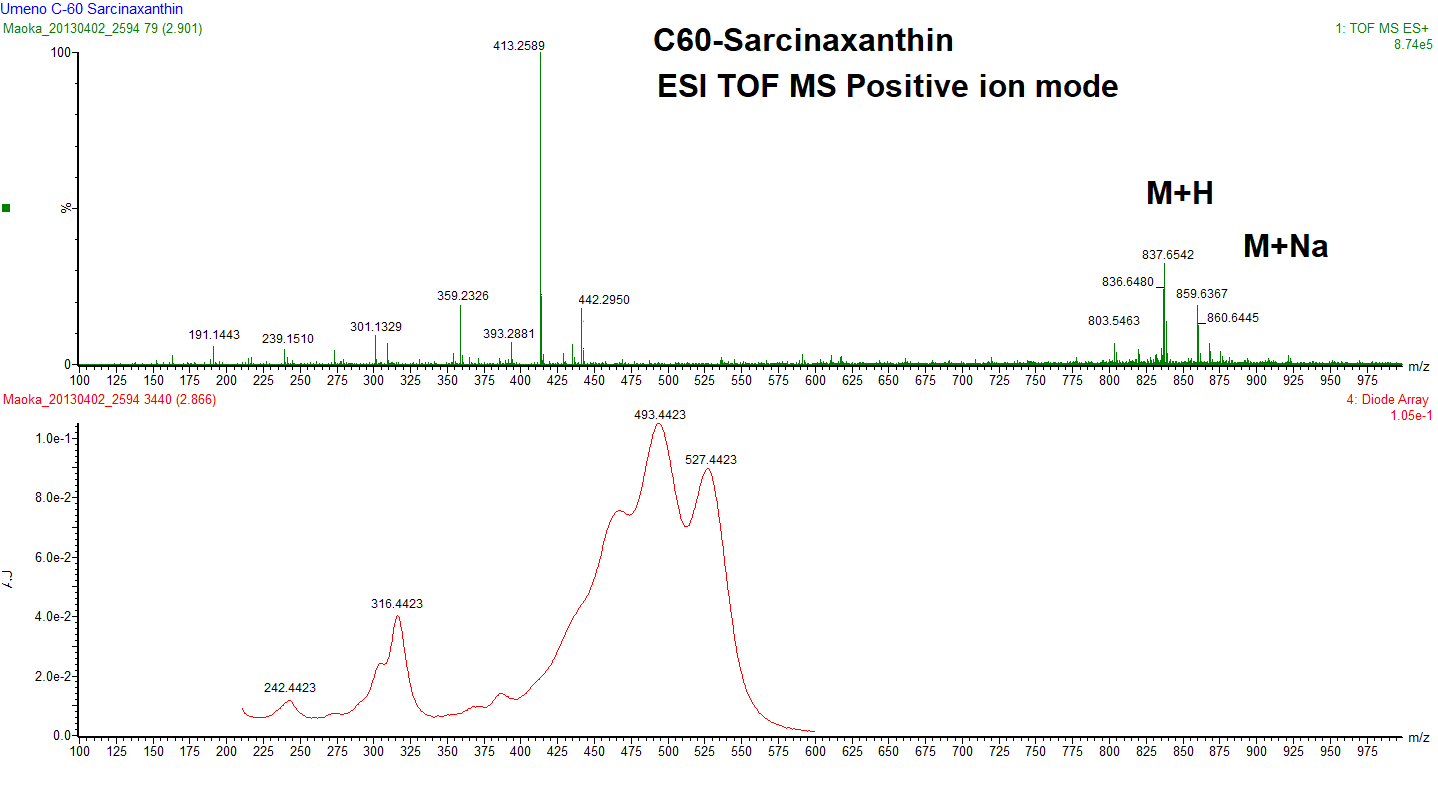


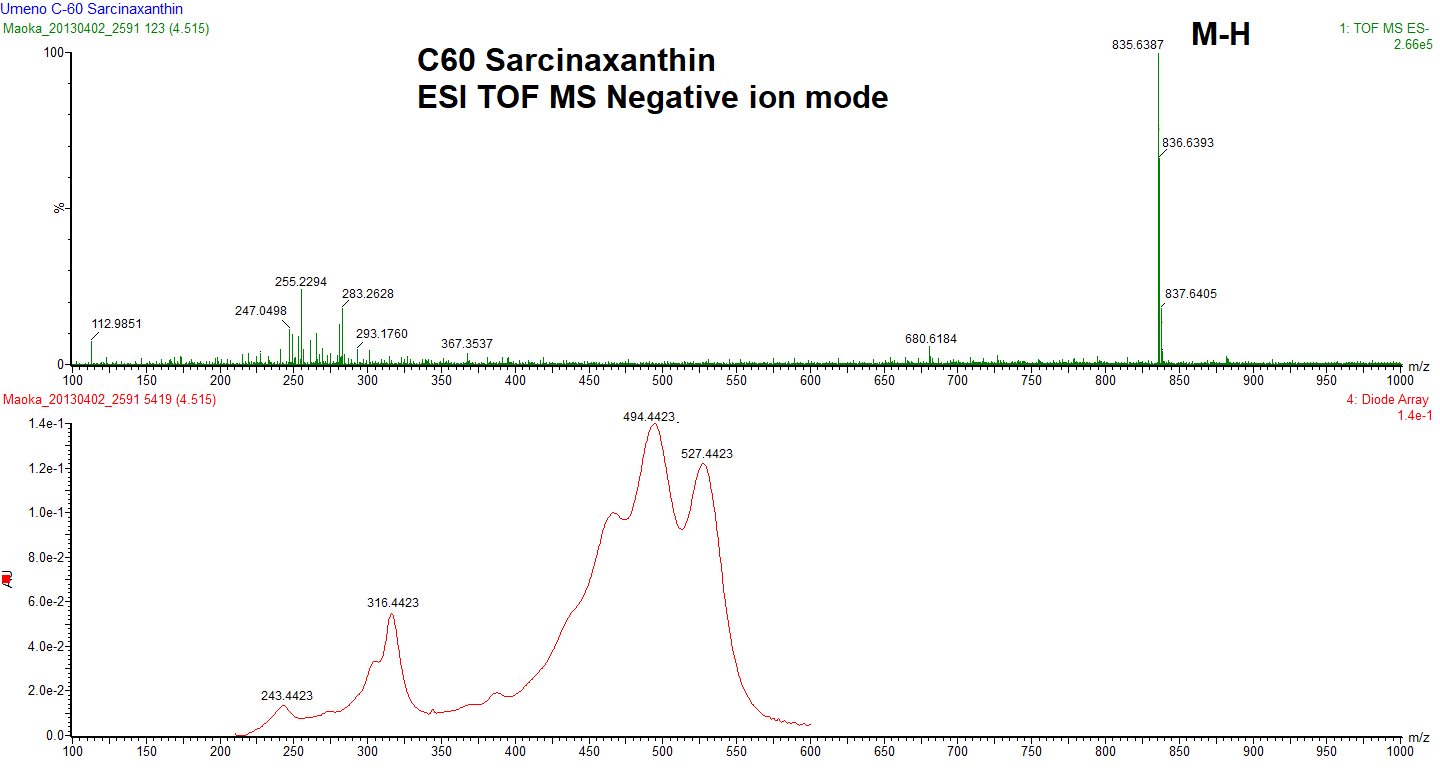


(continued to next page)

(**Supplementary Figure 2** continued from previous page)


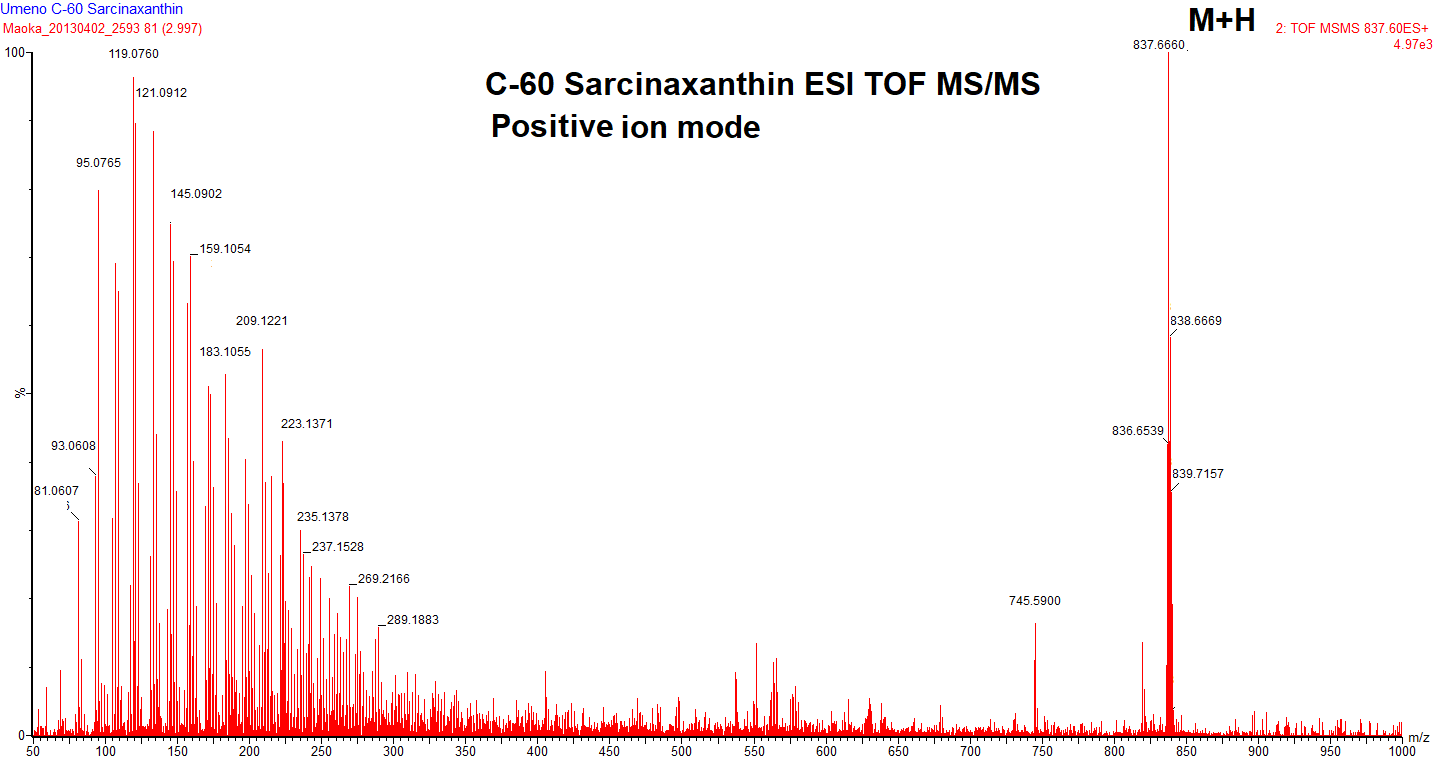


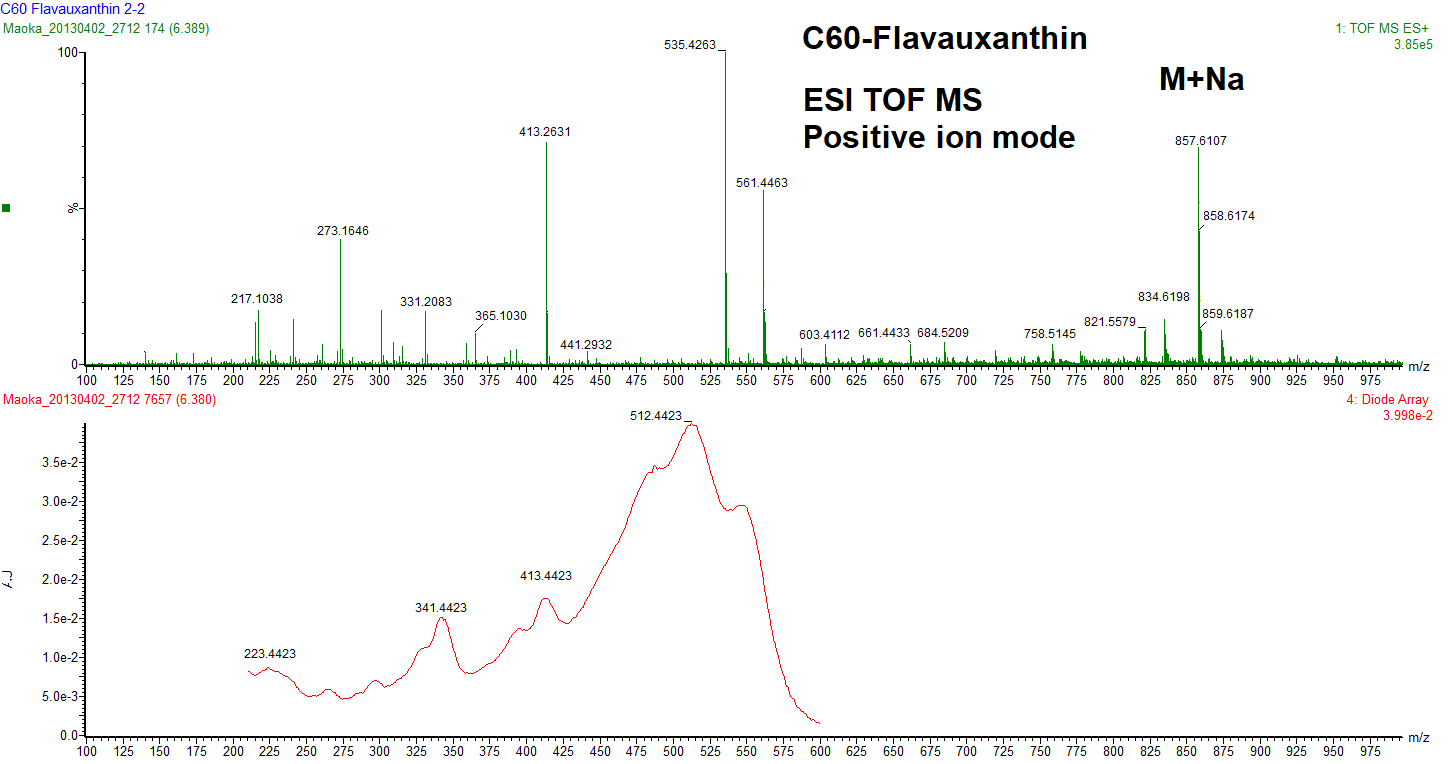


**Supplementary Figure 3.** Mass spectrum (ESI+) and absorbance spectrum for C_60_-flavuxanthin


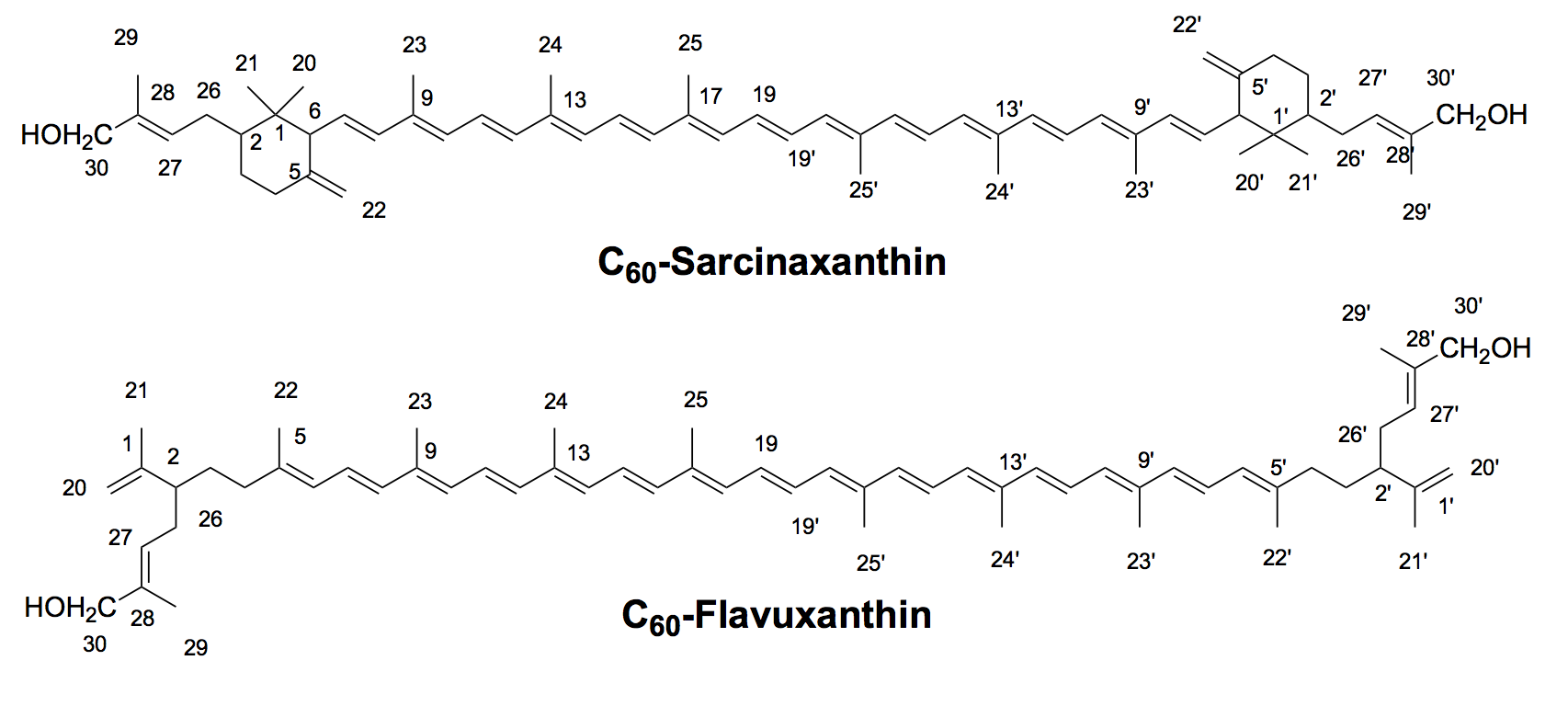


**Supplementary Figure 4.** C_60_-Sarcinaxanthin and C_60_-flavuxanthin structure numbering used for ^1^H-NMR in **Table 2.**

**Supplementary Table 1.** Strain and primers used to clone genes for elongases and cyclases.

Start and stop codons are indicated in capital letters. The annealing sequence is shown in italic.

| Strain | Primer name | Primer sequence |
| --- | --- | --- |
| *C. glutamicum*  (NBRC 12168G) | LL118-F | tttgggcccgtgtaggaggattacaaaA*TGatccctatcatcgatatttcacaaaatgagc* |
|  | LL118-R | tttactagt*TTAtatctgatgaattgctattagcagtatcgttatcac* |
| *C. efficiens* | LL120-F | tttgggcccgtgtaggaggattacaaaA*TGactgatttcctgatgtccttcatctatc* |
| (NBRC 100395) | LL121-R | tttactagt*CTAcacgaggtagacgctgatc* |
| *M. luteus* | LL122-F | tttgggcccgtgtaggaggattacaaa*ATGatccgcaccctcttctgg* |
| (NBRC 13867) | LL123-R | tttactagt*TCAgcgatcgtccgggtg* |
| *Dietzia sp*. CQ4 | LL124-F: | tttgggcccgtgtaggaggattacaaa*ATGatcgggctgtcctatctcc* |
| (NBRC 15352) | LL125-R | tttactagt*TCAggaccagacgagcaccgacca* |

**Supplementary Table 2.** The dna sequence of ORF and designed RBS

| ***M. luteus*** | |
| --- | --- |
| RBS of CrtE2 | gtcgacttatcaggaggaaatctac |
| CrtE2 | atgatccgcaccctcttctgggcgtcccggccggtcagctgggtgaacacggcctacccgttcgccgccgccgcgatcctgaccggggggctgcccgcgtggctggtggtcctgggcgtcgtgttcttcctcgtgccctacaacctggccatgtacggcatcaatgacgtgttcgacttcgcctcggacctgcgcaacccccgcaaggggggcgtggagggctccgtgctgggcgaccccgcggtgcgccgccgggtgctggcgtggtcggtgctgctgcccgtgccgttcgtggccgtgctcgcgggctggtccgccgtgcggggcgagtgggccgccgtgctggtgctcgcggtgagcctgttcgcggtggtggcgtactcctgggcggggctgcggttcaaggagcggcccttcctggacgccgcgacctccgccacccacttcgtctcccccgcggtctacggcctcgcgctggccggggcgacccccacgcccgccctggcggcgctgctgggggcgttcttcctgtggggcatggcctcgcagatgttcggggcggtgcaggacgtggtgccggaccgggaggggggcctggcctcggtggccaccgtgctgggcgctcggcgcaccgtcctgctcgccgccggcctgtacgcggcggcgggcctgctgctgctggccaccgacccgccgggcccgctcgcggcgctgctggccgtgccctacgtggtgaacaccctgcgcttccgccgcatcacggacgccacctcgggcgcggcccaccgcggctggcagctgttccttccgctgaactacgtgaccggcttcctcgtgaccctgctgctgatcgggtgggcgctgacccggggtgcagcagcatga |
| RBS of CrtYg | gtctacgaattaattaaggaggacatcgat |
| CrtYg | atgggtgctgaccctgggggaggaggcatgatctacctgctggccctgctgggtgtcatcggctgcatgctgctggtggaccggcgcttcgagctgttcctgtggcatcgcccgctcccggcgctgctggtgctggccgccggggtggcctacttcttcgcctgggacctgtgggggatcgccgaaggcgtgttcctgcaccggcagtcgccctacatgaccggggtgatgctcgccccccagctgcccctggaggaggggttcttcctgctcttcctcagccagatcacgatggtgctgttcaccggggcgctgcgcctgctgcgcggccggcgaggtgacgcccgtgccgcgacgccggccgatccgacagatgggggcagccggtga |
| RBS of CrtYh | agctttgttaaatctaggaggacggtacc |
| CrtYh | atgaccttccttgatcttgttctcgtcttcgtgggcttcgccctggccgtgctcgtgggcgccgccctcgtcggccgcgtgcggggcgagcacctgcgggccgtggcggccaccctggtggccctgtgggccctcacggcggtcttcgacaacgtgatgatcgccgcggggctcttcgactacggccatgagctgctggtgggtgcctacgtgggccaggcgcccgtggaggacttcgcctacccgctcggctccgccctgctgctgccggcgctctggctgctgctgacgagccgtggtcgtgccggtcggcgcggccctcggccgggacgccgtccccacccggatgatcgttga |
| ***C. efficiens*** | |
| RBS of CrtEb | gggccccataaggaggaataagaa |
| CrtEb | atgatggacacgatccgtgtggtgttctcctcctcccgccccatcagctgggtcaacaccgccttcccctacggcctggcctacctcctcgggggtggcagtatggactggctgttctgggtgggtgtgatcttcttcctcatcccctacaacatcgcgatgtacgggatcaatgatgtcttcgactatgaatcggatatccgcaacccccgcaagggtggtgtcgagggcgctgtggtgccgaagagataccaccccacgctgctgtgggcgtcgtcgatcaccaccatccccttcctggtggtgctctacgtcgccggcacctggatgtccacactgtggttgaccatctcggtgttcgcggtcatcgcctattccgcggcgggcctgcggttcaaggaacgtccgctgctggacgcgatcacctcctccacccacttcacctccccggcgctggtcggtgcgaccatcacgggtgccgatatctccaccgcgatgtggctcgccctggggtcgttcttcctgtggggcatggccagccagatcctcggggcggtgcaggatgtgcgtgccgaccgcgaggccggcctatcctccatcgccaccgccatgggtgcccgcggcgccgcacgactggccacgatgctctacctcgccgccgccgccctggtgttcctgctgccctcacccgcgtggatcgtcggcaccgccgcgttgacctatgtgctcaactccggtcgtttctggaacatcaccgatgacacctgcgaaagcgccaaccgtggctggaaggtcttcctctggttgaactacctggtcggcgcggtggtgtccatgaccctgatcagcgtctacctcgtgtag |
| RBS of CrtYe | gggtattttaggaggatatcgat |
| CrtYe | atgactgatttcctgatgtccttcatctatctgggttcgctgatcgtcttcatcggctgcatggtgttgtgtgaccaccggtggaagctcggcttcttccgcaacgcaccccgtgccgccctggcgatcggtgtcacctatgtcggattcctgctgtgggatgtgctcggcattgtcaccggcaccttctaccgtggtgattccccctatatgacgggcatcgacttagctccccacatgccgattgaggagctgttcttcctgttcttcctgtgctacctcaccctgaatctgacctccgcggtcagcctggtgttgaaaaccccgctgcctgagcagcggggggccaccacccagcccgccaataccggcgcaaccaccggggaggcccagccatga |
| RBS of CrtYf | agcttacccgagataaggaggaaggtacc |
| CrtYf | atgacctatgtcctcatgagcatccccttcctggtgttcgcactcgtggccttcgtgctcaaacgggccaacggcaccaggcaaatcacctgggtcaccctgctgaccaccctcatcctgttcgtgttgacgatcatcttcgacaacatcatggtgtgggccgatttcttcggttacggcgacacccagcacttaggcatctggatcggcctcatcccgatcgaggacctcttctatccgctgttcgctgccctgctgatccccgcgatctggttgccgggcaaactgtggcgcaggaacaccaccaccgcggacacatcttctgacgctgaaagggcataa |
| ***C. glutamicum*** | |
| RBS of CrtEb | gggccccggaaggaggacataacg |
| CrtEb | atgatggaaaaaataagactaattctattgtcatctcgccccattagctggatcaataccgcctacccctttggtctggcctacctattaaatgcaggagagattgactggctgttttggctaggcatcgtattttttcttatcccgtataacatcgccatgtatggtatcaacgatgtttttgattacgaatctgatatgcgtaatccccgcaaaggcggcgtcgagggggccgtgctaccgaaaagttcccacagcacactgttatgggcctcggctatctcaacaattcctttcctagttattcttttcatatttggcacctggatgtcgtctttatggctgacactctcagtgctagcagtgattgcttattcagcaccgaaattgcgttttaaagaacgcccctttatcgatgctctaacatcttctactcacttcacttcacctgcattaatcggtgcaacgatcactggaacatctccttcagcagcgatgtggatagcactgggatcctttttcttgtggggcatggccagtcagatccttggagcagtacaggatgttaatgcagaccgggaagctaatctgagctcaattgccactgtaattggggcgcgtggagccattcggctttcagtagtactttatttactagctgctgtgttagtcactactttgcctaatccggcgtggatcatcgggattgcgattctaacttacgtatttaatgccgcacgattttggaacattacagatgccagttgtgaacaggctaatcgcagttggaaagttttcctgtggctgaactactttgttggtgctgtgataacgatactgctaatagcaattcatcagatataa |
| RBS of CrtYe | gcgcccctatcaagcgtaggaggaagtcgac |
| CrtYe | atgatccctatcatcgatatttcacaaaatgagcaagatagcgatatttttatggcctttatttatctaggtactctcctagttctcattgggtgcatggctttgtgcgaccaccgttggaagctagcgttcttccgccatccgttacgagcaattctttcggtaggtgctgcatatattggatttcttttatgggatatatttggcattattactggcactttttatcgcggagactcagcgtttatgtccggtattaaccttgcaccccatatgcccattgaagaactttttttcttattcttcctctgctacatcaccctcaaccttacctcggcagcagcattatggcttaaagcaccactgcctaaaaaacccggtaaaaagtctcccctcacaccacagcgcgatactttccaaccaactaccactcccgaggttgaaccatga |
| RBS of CrtYf | agcttgagttcccatcaggaggagggtacc |
| CrtYf | atgacttatatttttataagcattccttttttagcaatagccatggtcctatttgtcttaaagctgcagtctggaacacctaaacttttaccaatcaccgctgtcagtgcccttaccctatgttccctaactatcatatttgataacctcatggtttgggctgatctctttggatatggcgatacccagcaccttggcatttggctcggtttaatccccctagaggatcttttctatccgctcttcgcagtacttctgattcctgccctatggttgcctggaaatatgtttaaacgcaggaaaaaacgtccacaccattccttacccaccatcgccaatggaagcatcactactagatccaccaccacgcaatctgagccagaaaagccgtag |
| ***Dietzia* sp.** | |
| RBS of LbtBC | aagctttgtcaggaggatacggct |
| LbtBC | atgacaagtctgtacactacactcaacctcaccatgtccatccccgtggtggccgtggccctcctcgcggcctggcgcctgcgcgggcccgagagacgccggtggatgatcggggtcggcggcgcgctgctgatcctcatgatcctcaccgcggtcttcgacaacatcatgatctcggccgggctcgtggcctatgacgactcgctcaccagcggcatccgcctgggtgtggcgcccatcgaggacttcgcctacgccgtggccgcggcggtcttcgtgccgtcggtgtgggcgttgctcaccgcgtcaccccgggtcggggccgaggtggggagcccgacggtgtcggggaggggggacgcgctgctgacgcgcgccccggagcccggcgacgacgacgaggtccgcacccccgagcggcccgggacgccgggactgctcaccacgctgttctggtcgtcccggccggtgtcgtgggtcaacaccgccgcgccgttcgccctggcgtacttcctggccaccggggggttcgacctcgtcggcgtgatcggcacgatcttcttcctggtgccgtacaacctcgcgatgtacgggatcaacgacgtcttcgactacgagtccgacctgcgcaacccccgcaagggcggggtcgaggggtcggtgctcgagcggtcccgtcacacggccaccctcgtggcctcggcggtgaccaccgtgccgttcctcgtgtacctggtgctgacggggacggtggagtcgtccctgtggctcgccgcgagcgcgttcgcggtgatcgcctacagcgccaaggggctgcggttcaaggagatcccgttcctggactcgctcacctccgcgttccacttcgtctccccggcgatcgtcggctggaccatcgccggtgcggacctgaccgggggggtgtgggcgtgcctgatcgcgttcatgctgtggggtgcggcctcgcaggccttcggggccgtgcaggacgtccgcttcgaccgcgaggcggacctgaagtccgtcgccaccgtgctcggggcccgggccgcggtgtggttcgcgctggcctgctacgccgcggcggtggtcgtcctgttggcggcggcgccgtggccggcctcgggggccgccttcgcgatcctgccgtacctcgccaccgtggcggtctatgtcggggtcaccgacgccgatgccgagcgcaccaacgagggctggaagcgcttcctggtgctcaacatgctggcgggcttctgcgtgacgcagatcgttctgtggtctgttcttgtctggtcatga |
| RBS of LbtA | atcttcgcccgcagaggaggaaatcgat |
| LbtA | atgatcgggctgtcctatctcctggtccaggtggtctccttcgccgggatcctggtgatcgaccaccgctggaagctggcggcgttccgtgcgcccgccgccgcggcgctcgccgtctccgcctccgtggcgttgctgctcacctgggacgtgctcggggtgcgcagcggggtgttcttccgcggacagaccgacttcatgaccgggctgctggtggcgccggagatcccgttcgaggaggtcgtgttcctggcgttcctgtcccacctcgcgctggtgtgcgcggcgggggtctcgcgcgcggtcgaccacgcgcgggactcccgggcggccagggcgtcccggccctcccggatgacgggtgaacgtcgttga |
